# Comparison of remote and local postconditioning against hepatic ischemic-reperfusion injury in rats

**DOI:** 10.1590/ACB360101

**Published:** 2021-02-01

**Authors:** Edson Yuzur Yasojima, Robson José de Souza Domingues, Renata Cunha Silva, Luis Fernando Freitas de Sousa, Sérgio Cunha Trindade

**Affiliations:** 1 PhD, Associate Professor. Universidade do Estado do Pará – School of Medicine – Department of Experimental Surgery –Belém (PA), Brazil.; 2 PhD, Associate Professor. Universidade do Estado do Pará – School of Medicine – Department of Experimental Surgery – Belém (PA), Brazil.; 3 Graduated student. Universidade do Estado do Pará – School of Occupational Therapy – Postgraduate Program in Surgery and Experimental Research – Belém (PA), Brazil.; 4 Graduate student. Universidade do Estado do Pará – School of Medicine – Department of Experimental Surgery – Belém (PA), Brazil.

**Keywords:** Ischemia, Reperfusion Injury, Ischemic Postconditioning, Antioxidants, Rats

## Abstract

**Purpose:**

The aim of this study is to compare the hepatic protective effect of both remote and local postconditioning (POS).

**Methods:**

Twenty-eight Wistar rats were assigned into four groups: sham group(SHAM), ischemia-reperfusion group (IR), local ischemic POS group (lPOS) and remote ischemic POS group (rPOS). Animals were subjected to liver ischemia for 30 min. Local ischemic POS group consisted of four cycles of 5 min liver ischemia, followed by 5 min reperfusion (40 min). Remote ischemic POS group consisted of four cycles of 5 min hind limb ischemia, followed by 5 min hind limb perfusion after the main liver ischemia period. After 190 minutes median and left liver lobes were harvested for biochemical and histopathology analysis.

**Results:**

All the conditioning techniques were able to increase the level of bothglutathione reductase and peroxidase, showing higher values in the rPOS group when compared to the lPOS. Also, thiobarbituric acid reactive substances were higher in all intervention groups when compared to SHAM, but rPOS had the lower rates of increase, showing the best result. The histopathology analysis showed that all groups had worst injury levels than SHAM, but rPOS had lower degrees of damage when compared to the lPOS, although it was not statistically significant.

**Conclusion:**

Remote postconditioning is a promising technique to reduce liver ischemia-reperfusion injury, once it increased antioxidants substances and reduced the damage.

## Introduction

The ischemia-reperfusion syndrome (IRS) is initiated by reestablishment of blood flow to ischemic tissues^1^ and, although this is a necessary step, it is characterized by tissue degeneration due to exaggerated production of reactive oxygen species (ROS) that lead to cell damage and to systemic inflammatory response. However, it is important to emphasize that there is no effective treatment against this illness^2^-^4^.

Many treatments have been studied along the years in order to mitigate the damage caused by this syndrome. Among those, there is ischemic conditioning, which is made of alternating and short cycles of ischemia and reperfusion before, during or after the obstruction procedure, once this method is efficient in reducing injury in several organs^5^-^8^.

Conditioning can be applied locally in the ischemic tissue, decreasing the damage caused by IRS^9^. Moreover, it can be also applied in a different tissue, known as remote ischemic conditioning. This procedure was first described by McClanahan *et al*.^10^, who verified that renal ischemia and reperfusion cycles created myocardium protection against IRS^11^,^12^.

The ischemia-reperfusion injury leads to several negative outcomes in the clinical context, as increased rates of morbidity and mortality and difficulty of postoperative recovery^13^. The liver is a highly oxygen dependent organ, what makes it more susceptible to hypoxia and anoxia damage. This fact explains the higher relevance of IRS in this organ to clinical practice, once this injury compromises liver function and promotes difficulties after surgical procedures, as transplantation and surgical resection^14^,^15^.

Several factors are important and contribute to hepatic ischemia-reperfusion injury, as Kupffer cells activation, oxidative stress and proinflammatory cytokines signaling^16^. Those elements are crucial to the amount of existing pathophysiological mechanisms of this injury, what hampers the development of methods of intervening in mediators that cause this problem^17^.

On the other hand, the ischemic postconditioning (POS) described by Zhao *et al*.^18^ is an easily applied technique in unexpected cases of ischemia, differently of preconditioning, showing more beneficial effects in reducing the damage caused by IRS in several clinical contexts, both locally and remotely, as it decreases hepatic tissue injury. However, the mechanism responsible for this hepatic endogenous protection is still unknown. It has also been observed reducing in renal, intestinal and cardiac injury by this technique^19^-^22^.

Thereby, the aim of this research is to compare the hepatic protective effect of local and remote postconditioning, analyzing which of those techniques is more beneficial to the IRS treatment.

## Methods

The research was approved by the Animal Use and Care Committee of the Universidade do Estado do Pará (No. 34/18). All experiments were performed in accordance to Brazilian law for scientific use of animals (Law: 11.794/08) and the National Institutes of Health guide for care and use of laboratory animals (NIH publications No. 8023, revised 1978).

Twenty-eight Wistar male rats (8-10 weeks), weighing 120-200 g, were obtained from the Evandro Chagas Institute. The animals were maintained at individual cages, at 22 °C, under a 12 h of light/dark cycle and allowed free access to water and standard chow. All surgical procedures and analysis were performed in the Laboratory of Morphophysiology Applied to Health.

### Experimental groups

The animals were randomly assigned into the following five groups (n = 7 for each group):

- Sham group (SHAM): In this group, the following surgical procedure was performed, but no liver ischemia was induced.
- Ischemia-reperfusion group (IR): In this group, liver ischemia was induced for 30 min, followed by reperfusion without conditioning.
- Local ischemic POS group (lPOS): 30 min of hepatic ischemia was followed by 40 min of autologous POS (four cycles of 5 min hepatic perfusion was followed by 5 min of hepatic ischemia).
- Remote ischemic POS group (rPOS): In this group, 30 min of hepatic ischemia was followed by40 min of remote POS. This technique consisted of four cycles of 5 min hind limb ischemia followed by 5 min hind limb perfusion, starting after the 30 min of hepatic ischemia. Hind limb ischemia was achieved by using an elastic rubber band tied around the thigh of the left leg^23^.

### Surgical procedures

After anesthetic application, using an intraperitoneal injection of ketamine hydrochloride 10% (70 mg/kg) and xylazine hydrochloride 2% (10 mg/kg), the animals were placed in supine position. Firstly, it was performed a median laparotomy in order to view the hepatic lobes. Then, the portal triad was isolated, and the left hepatic artery delicately dissected from the adjacent tissues, being occluded by microsurgical clamp application, leading to left and median lobe liver ischemia for 30 min^22^.

After the liver ischemia and conditioning protocols, the animals remained in reperfusion under surgical anesthesia for 2 h, and the left and median lobes were harvested for biochemical and histopathology analysis. Subsequently, the animals were euthanized by lethal anesthetic doses^24^.

### Biochemical analysis

The samples were homogenized in a 0.9% saline solution in a 1:1 ratio, and then immediately centrifuged at 4000 rpm for 10 min. After centrifugation, samples were directly transferred to Eppendorf tubes and stored at-80 °C until assayed. Thiobarbituric acid reactive substances (TBARS; mg/ml), glutathione peroxidase (GPx; mIU/mL), glutathione reductase (GR; mIU/mL) and catalase (CAT; IU/mL) levels were determined.

### Glutathione peroxidase and glutathione reductase

Glutathione peroxidase and glutathione reductase activities were measured by following the changes in nicotinamide adenine dinucleotide phosphate (NADPH) absorbance at 340 nm^25^. To calculate GPx and GR activities, extinction coefficient values established for NADPH were used.

### Catalase

Catalase was measured by the decomposition rate of H_2_O_2_ in the sample at 230 nm^26^. To calculate CAT activities, extinction coefficient values established for H_2_O_2_ were used.

*Thiobarbituric acid reactive substances*

Thiobarbituric acid reactive substances levels in liver tissues were analyzed by a method based on the reaction with thiobarbituric acid at 90-100 °C. In the thiobarbituric acid test reaction, malondialdehyde (MDA) or MDA-like substances and thiobarbituric acid react together to produce a pink pigment with a maximum absorption of 532 nm^27^.

### Histopathology analysis

After the median and left lobes resection, the median lobe was rinsed with saline solution and then stored in a solution of 10% formaldehyde. After this process, the hepatic segment was washed with water, cleaned with xylene and soaked in paraffin. Posteriorly, sections of 5 μm of paraffin were cut using a microtome and dewaxing. The cuts were stained with hematoxylin-eosin and analyzed with an optical microscope by a pathologist in a blind test^28^.

The levels of cell damage were assessed according to Takeda *et al*.^29^ criteria, being classified in: level 0 (without histological injury); level 1 (centrilobular congestion); level 2 (centrilobular congestion and hepatocytes degeneration in one or two central veins); level 3 (multifocal centrilobular congestion and portal hepatocytes degeneration).

### Statistical analysis

Statistical analysis was performed using the software *BioEstat 5.3*. All data were as expressed as means standard ± deviation. Shapiro-Wilk test was applied to confirm Gaussian distribution of the data. One-way analysis of variance with t-test and *post hoc* was used to assess differences between groups. Chi-square test was used for the histopathology analysis. Statistical significance was considered at p < 0.05.

## Results

**Figure 1:**
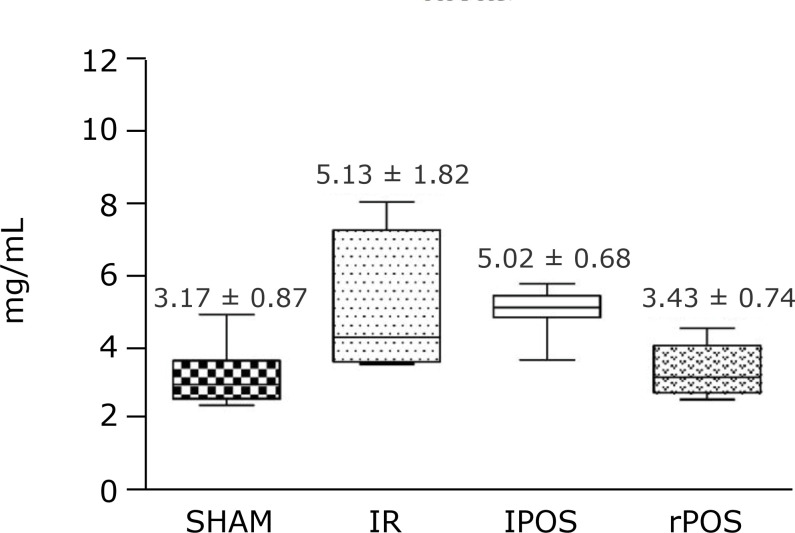
Thiobarbituric acid reactive substances concentration in hepatic tissue according to groups.One-way analysis of variance, t-test and *post hoc* test. Mean and standard deviation. P < 0.01 rPOS vs. IR and lPOS.

All tissue conditioning techniques were able to reduce the hepatic tissue MDA and MDA-like substances level. However, there was a statistically significant reduction with remote postconditioning (3.43 ± 0.74; p < 0.01 rPOS vs. IR and lPOS) (Fig. 1).

**Figure 2:**
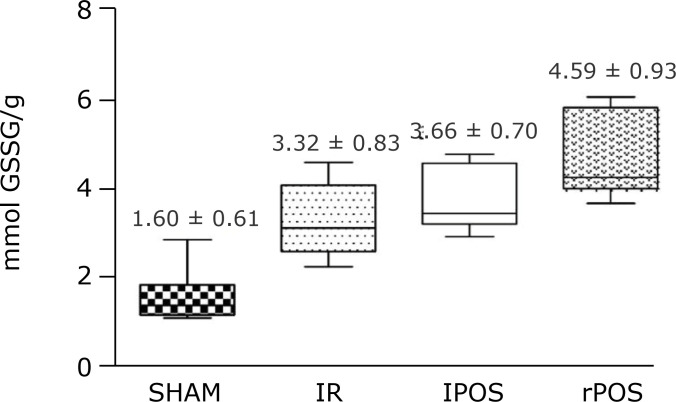
Activity of glutathione peroxidase in hepatic tissue according to groups. One-way analysis of variance, t-test and *post hoc* test. Mean and standard deviation. P < 0.03 rPOS vs. lPOS, p < 0.005 rPOS vs. IR, p < 0.0001 rPOS vs. SHAM.

**Figure 3:**
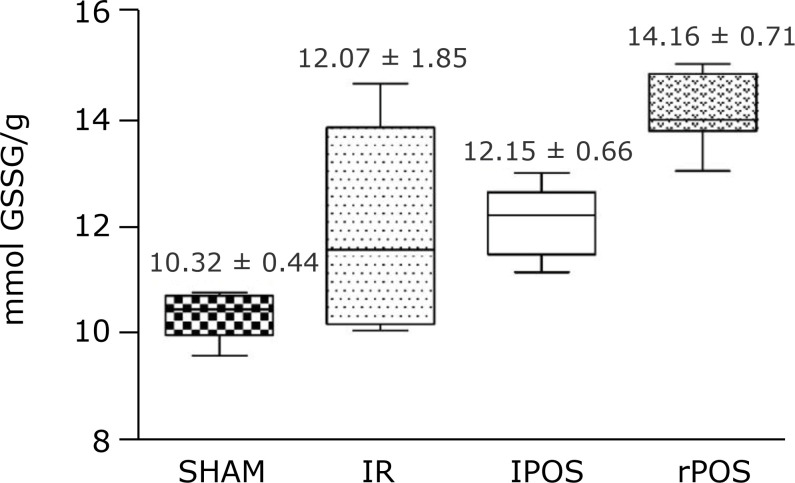
Activity of glutathione reductase in hepatic tissue according to groups. One-way analysis of variance, t-test and *post hoc* test. Mean and standard deviation. P < 0.001 rPOS vs. SHAM, IR and lPOS.

**Figure 4:**
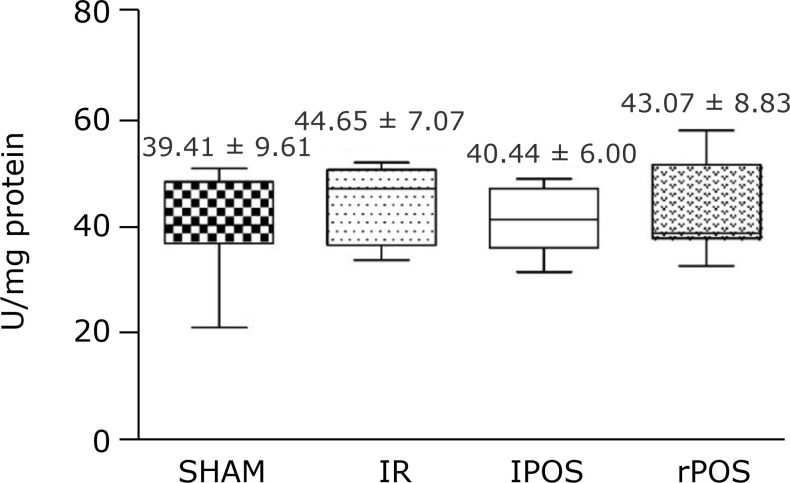
Activity of catalase in hepatic tissue according to groups. One-way analysis of variance, t-test and *post hoc* test. Mean and standard deviation. No statistical difference.

Furthermore, the rPOS protocol increased both glutathione peroxidase (4.59 ± 0.93; p < 0.03 rPOS vs. lPOS, p < 0.005 rPOS vs. IR, p < 0.0001 rPOS vs. SHAM) (Fig. 2) and glutathione reductase (14.16 ± 0.71;p < 0.001 rPOS vs. SHAM, IR and lPOS) (Fig. 3). There was no statistical difference between the groups in the analysis of catalase activity (Fig. 4).

The histology analysis of the groups demonstrated that there was a prevalence of level 2 injury in lPOS (p < 0.0001) and level 1 in rPOS (p = 0.1935), suggesting a better response of the remote technique, although there was no statistical difference among the groups (Table 1).

**Table 1:** Classification of the levels of cell damage according to groups. Chi-square adhesion test. P < 0.01 SHAM vs. IR, lPOS and rPOS; p < 0.01 lPOS vs. SHAM, IR and rPOS.

Classification	Groups							
		SHAM (%)		IR (%)		lPOS (%)		rPOS (%)
Level 0*		6.00 (85.7)		0 (0.0)		0 (0.0)		0 (0.0)
Level 1		1.00 (14.3)		2 (28.6)		1 (14.3)		4 (57.1)
Level 2**		0.00 (0.0)		3 (42.9)		5 (71.4)		3 (42.9)
Level 3		0.00 (0.0)		2 (28.6)		1 (14.3)		0 (0.0)
p-value		< 0.0001*		0.1845		< 0.0001**		0.1935

## Discussion

Ischemic postconditioning (IPOS) is a technique that focuses on the early events of reperfusion injury rendering the mitochondria and cell more tolerant to the perturbation caused by the ischemic-reperfusion injury, once the short repetitive cycles of this method maintain several protective endogenous substances inside the liver. Moreover, it seems to be a more suitable alternative for ischemic preconditioning, as long as it can be applied precisely in patients with unpredictably periods of inflow occlusion^30^. The new advent of remote ischemic conditioning also demonstrated a higher protection of liver injury, being a minimally invasive and low-cost technique, which can be associated to IPOS^11^.

In order to measure the cell membrane injury, the MDA and MDA-like substances levels demonstrated that the rPOS was the only technique able to reduce the oxidative stress, being statistically superior to lPOS. Although other studies reported that IPOS in the local organ could decrease those levels as consequence of a reduced oxidative stress^31^, the data in this study presented that only the remote technique was able to increase the antioxidant activities due to the inferior TBARS levels.

Furthermore, this data corroborates that fact, once the antioxidant substances of the liver, represented by glutathione peroxidase (GPx) and glutathione reductase (GR), both composing a redox system that combats ROS and xenobiotics in the cell, where increased significantly in the rPOS group when compared to lPOS^32^. Those data allowed to detect that the remote technique had better outcome in protecting the liver from oxidant injury due to increasing of protective substances, which is also demonstrated by other studies^33^.

On the other hand, other vital antioxidant enzyme released after liver ischemic-reperfusion injury is catalase. This study could not demonstrate the increase levels of this enzyme in any tissue conditioning technique due to the absence of significant statistical analysis of liver samples. More studies should be made to testify those findings.

From the tissue histological analysis, it was detected that all ischemic groups, regardless the conditioning technique or its absence, had higher levels of tissue damage when compared to SHAM. The data presented that lPOS group had classification level 2 as the most prevalent one, meanwhile rPOS had level 1 as main degree of liver damage. Although there was no statistical significance when comparing both groups, these findings allows to suppose that remote postconditioning is slightly superior than local on preventing hepatic ischemic-reperfusion injury, due to the different degree of hepatic injury assessed in histology.

Other studies demonstrated the important role of POS - including remote technique - in reducing tissue damage of ischemic and reperfusion injury, showing better results in those groups and this technique ability of ameliorate the histological features of some organs, as brain and myocardium^34^,^35^. That being said, it is indispensable that more researches try to elucidate the POS - local or remote - function in reducing liver ischemic damage.

## Conclusions

Therefore, it was observed that rPOS is the most capable technique to improve the antioxidant defenses of the organism against an ischemia-reperfusion injury. In addition, this method might be the most promising way to reduce the histological damage of IRS.
